# Pregnancy Outcomes among Women Receiving rVSVΔ-ZEBOV-GP Ebola Vaccine during the Sierra Leone Trial to Introduce a Vaccine against Ebola

**DOI:** 10.3201/eid2603.191018

**Published:** 2020-03

**Authors:** Jennifer K. Legardy-Williams, Rosalind J. Carter, Susan T. Goldstein, Olamide D. Jarrett, Elena Szefer, Augustin E. Fombah, Sarah C. Tinker, Mohamed Samai, Barbara E. Mahon

**Affiliations:** Centers for Disease Control and Prevention, Atlanta, Georgia, USA (J.K. Legardy-Williams, R.J. Carter, S.T. Goldstein, O.D. Jarrett, S.C. Tinker, B.E. Mahon);; University of Illinois at Chicago, Chicago, Illinois, USA (O.D. Jarrett);; The Emmes Corporation (E. Szefer);; University of Sierra Leone College of Medicine and Allied Health Sciences, Freetown, Sierra Leone (A.E. Fombah, M. Samai)

**Keywords:** Ebola, pregnancy, Ebola virus disease, vaccine safety, Ebola vaccine, viruses, vaccine-preventable diseases, STRIVE, Sierra Leone, West Africa, rVSVΔ-ZEBOV-GP, Sierra Leone Trial to Introduce a Vaccine against Ebola, rVSVΔ-ZEBOV-GP, Ebola virus

## Abstract

Little information exists regarding Ebola vaccine rVSVΔG-ZEBOV-GP and pregnancy. The Sierra Leone Trial to Introduce a Vaccine against Ebola (STRIVE) randomized participants without blinding to immediate or deferred (18–24 weeks postenrollment) vaccination. Pregnancy was an exclusion criterion, but 84 women were inadvertently vaccinated in early pregnancy or became pregnant <60 days after vaccination or enrollment. Among immediate vaccinated women, 45% (14/31) reported pregnancy loss, compared with 33% (11/33) of unvaccinated women with contemporaneous pregnancies (relative risk 1.35, 95% CI 0.73–2.52). Pregnancy loss was similar among women with higher risk for vaccine viremia (conception before or <14 days after vaccination) (44% [4/9]) and women with lower risk (conception >15 days after vaccination) (45% [10/22]). No congenital anomalies were detected among 44 live-born infants examined. These data highlight the need for Ebola vaccination decisions to balance the possible risk for an adverse pregnancy outcome with the risk for Ebola exposure.

The 2014–2016 West Africa Ebola virus disease outbreak was unprecedented in magnitude and complexity, resulting in >28,000 cases and >11,000 deaths in the 3 highly affected countries (Sierra Leone, Guinea, and Liberia) (*1*). During the outbreak, clinical trials of the investigational Ebola vaccine rVSVΔG-ZEBOV-GP (Merck, https://merck.com) were rapidly implemented. The vaccine, a live-attenuated recombinant vesicular stomatitis virus (rVSV) vaccine, was found to be protective when used in a ring vaccination strategy in Guinea (*2*). This result spurred subsequent use of this vaccine under expanded use protocols as part of the public health response to Ebola outbreaks. As of late November 2019, >250,000 investigational doses had been administered in 2 outbreaks in the Democratic Republic of the Congo during 2018 and 2019 (*3*). The vaccine received conditional marketing approval from the European Medicines Agency and World Health Organization prequalification in November 2019 (*4*). However, little information on the safety of the vaccine for pregnant women is available, making decisions about vaccination during pregnancy challenging.

Pregnancy was an exclusion criterion for all rVSVΔG-ZEBOV-GP clinical trials, not only because so little was known about the safety of the vaccine generally but also because adverse effects on pregnancy were biologically plausible (*5*,*6*). A phase 1 trial was paused because of concerns about postvaccination arthritis associated with dissemination of the vaccine rVSV into the joints, raising concerns that other adverse reactions could occur consequent to vaccine viremia (*7*–*9*). In the Sierra Leone Trial to Introduce a Vaccine against Ebola (STRIVE) (*10*,*11*), some women were enrolled who were inadvertently vaccinated early in pregnancy, and some women became pregnant ≤60 days after enrollment or vaccination. STRIVE followed these women for pregnancy outcomes. We have previously reported preliminary analysis of pregnancy outcomes (*10*); we now report a more detailed analysis.

## Methods

**Figure 1:**
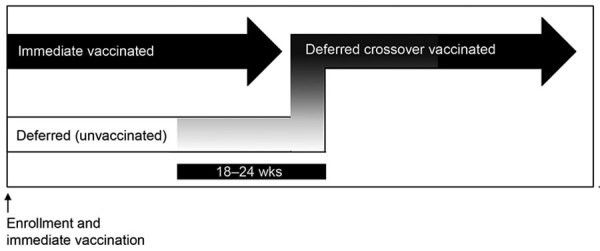
Enrollment and vaccination period for 84 participants in Sierra Leone Trial to Introduce a Vaccine against Ebola (STRIVE). Three participants randomized to the immediate group were unvaccinated. After vaccination, participants in the deferred group were eligible for vaccination at 18–24 weeks postenrollment. Upon vaccination, participants in the deferred group were referred to as the deferred crossover vaccinated group.

STRIVE was a phase 2/3, unblinded, individually randomized clinical trial to assess the safety and efficacy of rVSVΔG-ZEBOV-GP; the methods have been detailed previously (*10*). In brief, adult (>18 years of age) healthcare and Ebola frontline workers were randomized to immediate or deferred (18–24 weeks later) vaccination with a single intramuscular dose (nominal 2 × 10^7^ PFUs) of rVSVΔG-ZEBOV-GP vaccine. No placebo was used; all participants who were eligible for vaccination were offered vaccine by the end of the study. The immediate group was vaccinated from April through August 2015 and the deferred group from September through December 2015. Before the deferred group was vaccinated, they were referred to as the unvaccinated group; once vaccinated, they were referred to as the deferred crossover vaccinated group (Figure 1). 

Pregnancy was an exclusion criterion for the study and was assessed during preenrollment screening. At this screening, women of reproductive age (18–49 years of age) were asked if they were pregnant and were required to take a urine pregnancy test. Before deferred vaccination, women 18–49 years of age were again asked if they were pregnant and were required to take another urine pregnancy test. Vaccinated women were counseled to avoid pregnancy for <60 days after vaccination. Contraception was available locally but was not provided by the trial. Participants were referred to the Ministry of Health’s Family Planning Service Clinics if they requested contraception.

All study participants received monthly calls for 6 months after vaccination to monitor for the onset of Ebola, safety outcomes, and, for women, pregnancy. Home visits were conducted if participants could not be contacted by phone. A STRIVE telephone hotline was also available 24/7 for participants to report any medical issue. Women who reported pregnancy were monitored through their pregnancy outcome.

The Sierra Leone Ethics and Scientific Review Committee, the CDC Institutional Review Board, the Pharmacy Board of Sierra Leone, and the US Food and Drug Administration reviewed and approved the study design. The trial was conducted in accordance with the International Council for Harmonization’s Good Clinical Practice standards (https://ich.org). All women signed informed consent forms at screening for pregnancy testing and enrollment.

### Pregnancy and Infant Follow-Up

During each monthly follow-up call, we asked female participants about pregnancy. If a woman reported being pregnant, no confirmation (e.g., urine or blood pregnancy test, physical examination, or sonogram) was required. For the purpose of follow-up, we calculated the estimated date of conception (EDC) as the date of the last menstrual period (LMP) plus 14 days. Women whose EDC was <60 days after enrollment or vaccination (immediate or deferred crossover) were followed monthly by a STRIVE study nurse until a pregnancy outcome was documented. STRIVE did not provide clinical care for pregnant women; women were referred to free prenatal care provided by the Ministry of Health. For 1 woman whose LMP was not known and who delivered at full term, we used the actual delivery date minus 40 weeks to determine EDC. In this analysis, we include women whose EDC was <60 days after enrollment or vaccination.

We categorized pregnancy outcomes as live birth, pregnancy loss, ectopic pregnancy, or unknown. The live birth category included both preterm and term births, as self-reported by the mother. Because STRIVE did not routinely collect exact dates of pregnancy losses, we could not reliably differentiate between spontaneous abortions and stillbirths, so we grouped them into a single pregnancy loss category for the analysis. Induced abortion is not legal in Sierra Leone but is reported to be available (*12*); women who had an induced abortion might not have reported their pregnancy or might have reported the pregnancy outcome as a spontaneous abortion. The unknown category included women for whom follow-up was not completed and whose pregnancy outcome therefore could not be determined. After delivery, mothers were asked about the date of delivery and the infant’s birth weight.

A STRIVE study nurse examined live-born infants at >28 days of life. Study nurses received training on infant examination by a team of physicians from Sierra Leone and the United States that included a pediatrician. The infant examination, which was usually conducted at the woman’s home, included examination of the infant’s general appearance, extremities, head and face, chest, abdomen, anus, genitourinary system, and musculoskeletal system for the presence of external congenital anomalies; no vision or hearing tests were conducted. Nurses were instructed to refer infants to a study physician for further evaluation if the examination raised any concerns, and, if necessary, the infant was then referred to a pediatrician.

Using conservative estimates, we defined pregnancy groups as either high viremia risk (if EDC was before vaccination or <14 days after vaccination, including women who were pregnant at the time of vaccination) or low viremia risk (if the EDC was >15 days after vaccination). We based these determinations on studies showing that viremia or PCR positivity peaks in healthy adults 1–3 days after vaccination and usually resolves within 7–14 days after vaccination (*7*–*9*,*13*–*15*).

### Statistical Methods and Analyses

For comparison of outcomes in vaccinated and unvaccinated women, we included only pregnancies in the immediate vaccinated group and the unvaccinated group. We did not include the deferred crossover vaccinated group in this specific analysis because multiple time-related factors could have affected earlier (immediate and unvaccinated) and later (deferred crossover) pregnancies differently. These potentially confounding factors include differential access to healthcare services, such as prenatal and maternity services, during versus at the end of the Ebola outbreak; infections such as malaria that have strong seasonal patterns; and attitudes toward pregnancy during versus after the Ebola epidemic. For comparison of pregnancy outcomes based on viremia risk, however, we included both the immediate and deferred crossover vaccinated groups separately (because of the confounding we have described) and combined (because of small sample size).

We reported descriptive statistics summarizing maternal and infant characteristics. Counts and percentages are reported for binary characteristics; medians and ranges are reported for continuous characteristics. We computed the relative risk (RR) of pregnancy loss and the associated exact 95% CI, comparing the immediate vaccinated and unvaccinated groups. We used Barnard’s unconditional exact test to test for differences in occurrence of pregnancy loss between the immediate vaccination and unvaccinated groups and between viremia risk categories within vaccination groups. Women with unknown outcomes were excluded from primary analyses. However, we conducted sensitivity analyses assuming that all pregnancies with unknown outcome were classified as either pregnancy loss or live birth to understand the maximum potential effect of missing data.

## Results

Of the 8,651 participants enrolled in STRIVE, 3,101 were women of reproductive age (18–49 years of age). Eighty-four (2.7%) of these women had a singleton pregnancy (no multiple gestations) with EDC <60 days from enrollment or vaccination, including 31 in the immediate vaccinated group, 35 in the unvaccinated group, and 18 in the deferred crossover vaccinated group. At enrollment, the median age of these women was 28 years (range 20–40 years); most of these women were nurses (66 [79%]) or frontline Ebola responders (14 [16%]) (Table 1). Baseline demographic characteristics of vaccinated (immediate and deferred crossover) and unvaccinated pregnant women were generally similar.

**Table 1:** Demographic characteristics and pregnancy outcomes by vaccination group among 84 women with estimated date of conception <60 days from vaccination or enrollment, Sierra Leone Trial to Introduce a Vaccine against Ebola*

Characteristic	Immediate vaccinated	Unvaccinated	Deferred crossover vaccinated	Total
Total	31	35	18	84
Median age, y (range)	27 (22–38)	29 (20–40)	28 (20–38)	28 (20–40)
Primary occupation				
Nurse†	24 (77)	29 (83)	13 (72)	66 (79)
Frontline worker	5 (16)	6 (17)	3 (17)	14 (16)
Other‡	2 (7)	0	2 (11)	4 (5)
Prior pregnancy				
No	10 (32)	12 (34)	6 (33)	28 (33)
Yes	21 (68)	23 (66)	12 (67)	56 (67)
Pregnancy outcomes				
Known	31 (100)	33 (94)	17 (94)	81 (96)
Live birth	17 (55)	22 (66)§	12 (71)§	51 (63)
Preterm delivery	2	0	0	2
Term delivery	15	22	12	49
Pregnancy loss¶	14 (45)	11 (33)§	5 (29)§	30 (37)
Unknown	0	2 (6)	1 (6)	3 (4)

*Values are no. (%) except as indicated. †Includes nurse, nurse aide, maternal–child health aide, nursing student, midwife, community health nurse, and vaccinator. ‡Includes allied health profession, community health worker, dentist, medical counselor, nutritionist, physiotherapist, vaccinator, and surveillance worker. §Denominator for live birth and pregnancy loss includes number of pregnancies with known outcomes (i.e., unknown outcomes excluded). ¶Pregnancy loss includes spontaneous abortion and stillbirth.

The 84 pregnancies led to 51 live births (49 term and 2 preterm) and 30 pregnancy losses (Table 1). For 3 women (2 unvaccinated and 1 deferred crossover vaccinated), the pregnancy outcome was not known. No ectopic pregnancies or neonatal deaths were reported. Of the 51 live births, 29 were in vaccinated women and 22 in unvaccinated women. Most (46 [90%]) infants were delivered in a hospital; 5 (10%) were born at home. The median birth weight was 3,210 g (range 2,400–5,200 g). STRIVE staff obtained consent to examine 44 of the 51 infants (born to 28 vaccinated and 16 unvaccinated women); no external congenital anomalies were documented among these infants.

A total of 7 serious adverse events (SAEs) were reported among pregnant participants. Five SAEs were hospitalizations for a pregnancy-related complication: gestational hypertension (2 cases), prolonged labor (2 cases), and a postpartum hemorrhage (1 case) that resulted in a maternal death. Two pregnant women had hospitalizations for SAEs not related to pregnancy (1 for enteritis and 1 for malaria).

**Figure 2:**
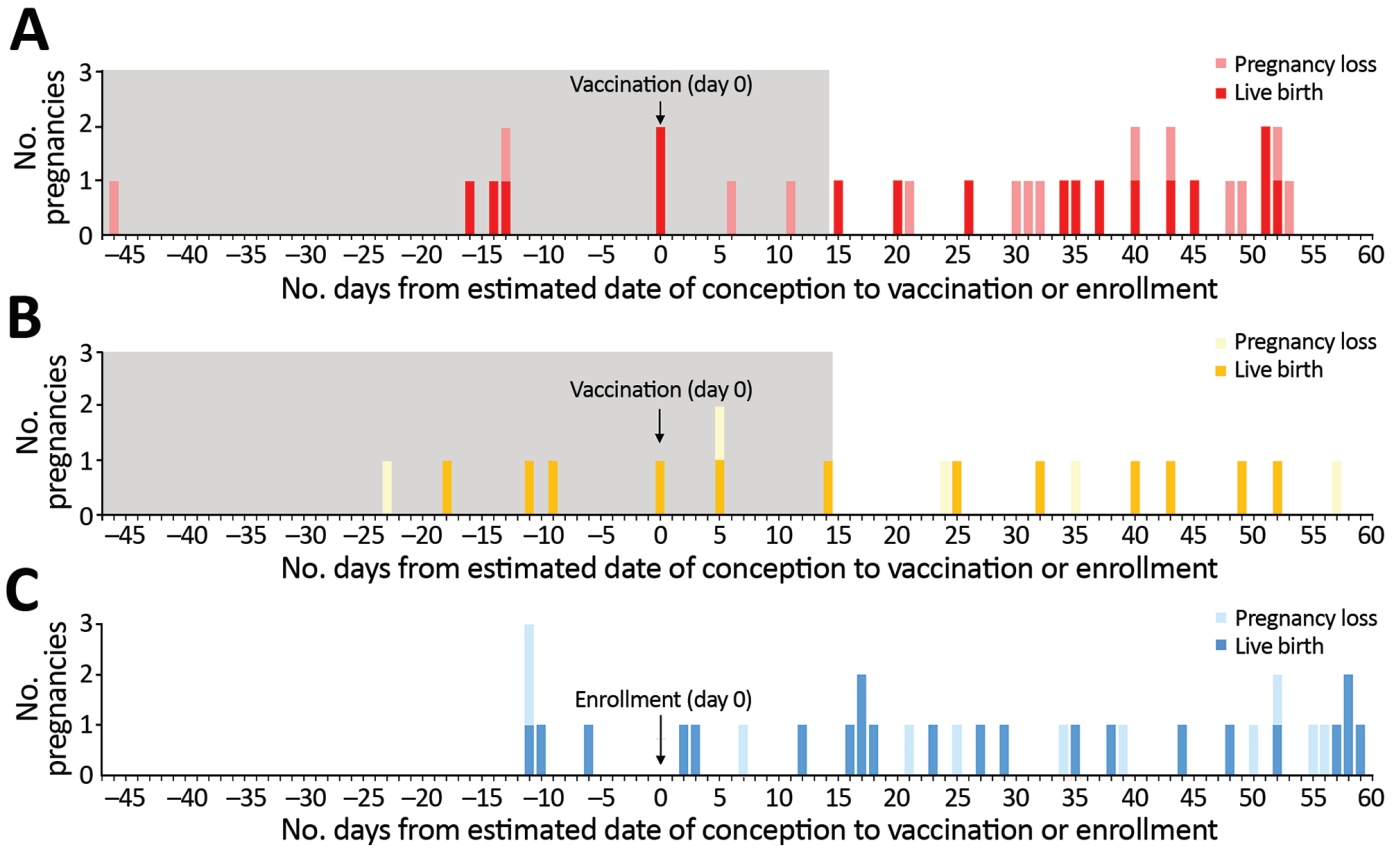
Number of pregnancies by estimated date of conception relative to vaccination or enrollment among 81 participants in the Sierra Leone Trial to Introduce a Vaccine against Ebola (STRIVE). A) Immediate vaccination group (n = 31). B) Deferred crossover vaccination group (n = 17). C) Unvaccinated group (n = 33). Because pregnancy outcome for 3 of the 84 women was unknown, these 3 women are not included in the figure. Outcomes include live birth (term and preterm) and pregnancy loss (early and late loss). Gray shaded area denotes the high viremia risk period (i.e., women who were pregnant when vaccinated or became pregnant 0–14 days after vaccination).

We compiled the number and outcomes of pregnancies by EDC among participants in this analysis (Figure 2). Among the 48 vaccinated women with a known pregnancy outcome based on EDC calculations, 9 were pregnant at the time of vaccination, all with a negative self-report and negative urine pregnancy test. An additional 8 women had an EDC of 0–14 days after vaccination. Thus, a total of 17 women were in the high viremia risk group. We observed no difference in proportions of live births and pregnancy loss between women who were pregnant when vaccinated and those who became pregnant 0–14 days after vaccination (data not shown). The other 31 women, with EDC 15–60 days after vaccination, comprised the low viremia risk group.

### Pregnancy Outcome by Vaccination Group

In the comparison of pregnancy outcome in the vaccinated and unvaccinated women with known outcomes, pregnancy loss occurred more frequently in the immediate vaccinated group (45% [14/31]) compared with the unvaccinated group (33% [11/33]), although this difference was not statistically significant (unadjusted RR 1.35 [95% CI 0.73–2.52]; p = 0.34) (Table 1). The calculated gestational ages were similar between vaccination groups (data not shown). A sensitivity analysis that included the 2 pregnancies with unknown outcomes as pregnancy losses reduced the RR to 1.22 (95% CI 0.68–2.17); including them as live births increased the RR to 1.44 (95% CI 0.77–2.68).

### Pregnancy Outcome by Viremia Risk Group

Pregnancy loss occurred at similar rates in the high viremia risk group (35% [6/17]) and the low viremia risk group (42% [13/31]; p value for comparison 0.69) and with similar patterns in the immediate vaccinated and deferred crossover vaccinated groups (Table 2). Within the high viremia risk group, pregnancy loss was reported for 3 (33%) of the 9 women who were pregnant when vaccinated and by 3 (38%) of the 8 women whose EDC was 0–14 days after vaccination. In the sensitivity analyses, including the 1 unknown pregnancy outcome in the deferred crossover group as a pregnancy loss or as a live birth did not change the results (data not shown).

**Table 2:** Pregnancy outcome by risk for vaccine viremia during pregnancy among 48 women with estimated date of conception <60 days from vaccination, Sierra Leone Trial to Introduce a Vaccine against Ebola*

Characteristic	Live birth	Pregnancy loss	Total	Barnard’s exact p value
Immediate vaccinated, no.	17	14	31	1
High viremia risk	5 (56)	4 (44)	9	NA
Low viremia risk	12 (55)	10 (45)	22	NA
Deferred crossover vaccinated, no.	12	5	17	0.75
High viremia risk	6 (75)	2 (25)	8	NA
Low viremia risk	6 (67)	3 (33)	9	NA
Total vaccinated, no.	29	19	48	0.69
High viremia risk	11 (65)	6 (35)	17	NA
Low viremia risk	18 (58)	13 (42)	31	NA

*Values are no. (%) except as indicated. NA, not applicable. †High viremia risk is defined as estimated date of conception before vaccination or 0–14 days after vaccination. Low viremia risk is defined as estimated date of conception 15–60 days after vaccination.

## Discussion

This analysis of STRIVE clinical trial data provides valuable, although not conclusive, information about pregnancy outcomes in women who were pregnant when they were vaccinated with the investigational Ebola vaccine rVSVΔG-ZEBOV-GP or who became pregnant within 60 days after vaccination. The 45% rate of pregnancy loss in the immediate vaccination group was not significantly higher than the 33% rate in the contemporaneous unvaccinated group. However, only a small number of pregnancies occurred among participants in STRIVE, and data from larger study samples would be needed to rule out a meaningful difference in the percentage of pregnancy losses. 

We observed no difference in pregnancy loss when we compared women who had a high likelihood of having been pregnant during the period of post-vaccination vaccine viremia to those who became pregnant later after vaccination. Also, 44 of 51 live-born infants were examined, and no external congenital anomalies were detected, although only a small number of infants were born to vaccinated women and no diagnostic testing was conducted. In the absence of more definitive data, our results can inform consideration of the inclusion of pregnant women in rVSVΔG-ZEBOV-GP vaccination programs, such as in recent and, as of December 2019, ongoing responses to Ebola outbreaks in the Democratic Republic of the Congo (*3*). The limitations of our results highlight the need for ongoing collection of information on pregnancy outcomes in vaccinated women.

The rVSVΔG-ZEBOV-GP vaccine has a vesicular stomatitis virus (VSV) backbone in which the gene encoding the VSV envelope glycoprotein is replaced with the gene encoding the Zaire Ebola virus (Kikwit strain) glycoprotein. VSV normally infects animals; human disease has been reported rarely, and no information exists regarding wild-type VSV infection in human pregnancy (*16*). In 1 study from the 1970s, spontaneous abortion and neonatal death were reported in ferrets experimentally infected with VSV-Indiana, a wild-type VSV strain, and virus was recovered from the placentas of 2 experimentally vaccinated ferrets (*5*). Vaccine VSV might be less virulent than wild-type VSV (*17*). Because rVSVΔG-ZEBOV-GP is a replication-competent vaccine, rVSVΔG-ZEBOV-GP vaccination commonly produces rVSV vaccine viremia within a few days after vaccination (*8*,*9*,*13*,*14*). The detection of vaccine virus in joint fluid and skin lesion in some vaccinated persons in phase 1 studies of this vaccine raised the possibility of adverse effects on pregnancy (*8*,*13*). A total of 20 additional women who were pregnant when vaccinated or became pregnant soon after vaccination with rVSVΔG-ZEBOV-GP have been reported in 3 clinical trials other than STRIVE during 2014–2016. Outcomes for these 20 pregnancies included 2 spontaneous abortions at ≈1 month after conception and 1 stillbirth, for an overall pregnancy loss rate of 15% (*7*,*18*,*19*).

In STRIVE, as in the other phase 2/3 rVSVΔG-ZEBOV-GP trials initiated during the West Africa Ebola epidemic, pregnancy was an exclusion criterion. STRIVE screened women of childbearing age for pregnancy and counseled vaccinated participants to avoid becoming pregnant for <60 days after vaccination. For most women who were inadvertently vaccinated while pregnant, conception was probably too recent for the pregnancy test to be positive or for the woman to realize she was pregnant. Use of a questionnaire, such as the Pregnancy Exclusion Checklist, in combination with the urine pregnancy test might have more effectively identified women who were early in pregnancy (*20*). However, the EDC of 1 of the women with a negative urine pregnancy test was 46 days before vaccination. This woman possibly did not know or did not disclose she was pregnant, or the urine pregnancy test might have been performed incorrectly or was not able to detect the pregnancy. A strength of our analysis was that the design of the trial yielded a contemporaneous unvaccinated group for comparison to the vaccinated group, and that information was available on the outcomes of almost all pregnancies, a result of the identification and comprehensive follow-up of all pregnant women in STRIVE. 

Our study had several limitations, however, beyond the small sample size and inability to adjust for confounding factors. In cases of pregnancy loss, information on the timing of the loss was often lacking, limiting our ability to differentiate between early and late pregnancy loss. Also, because we had limited information about the timing of pregnancies (ultrasound dating is rarely available in Sierra Leone), we had to calculate EDCs from LMPs, which is not an ideal method (*21*). Unrecognized or unreported pregnancies that led to pregnancy loss might have occurred, and because we did not confirm the pregnancies, a pregnancy loss might have been reported when a woman was not actually pregnant (i.e., late menstrual cycle reported as spontaneous abortion). Another important limitation is that STRIVE has no information on long-term outcomes in infants.

The few published data on pregnancy loss for Sierra Leone are limited to stillbirths (late pregnancy) (*22*) and do not include spontaneous or induced abortions. However, some conditions common in Sierra Leone, such as malaria, increase the risk for stillbirth and spontaneous abortion (*23*,*24*). A limitation of our data is that we were not able to ascertain the number of pregnancy losses in STRIVE that were caused by induced abortion. Induced abortions are illegal in Sierra Leone, but they occur (*12*). When induced abortions are included in analysis of US pregnancy outcomes, ≈34% of pregnancies end in loss, similar to the loss percentage observed during the STRIVE trial (37%) (*25*). Because the trial was unblinded, women in the immediate vaccinated group and the unvaccinated group knew their vaccination status, which could have affected their decision-making. For instance, vaccinated women might have been concerned about the safety of the vaccine in pregnancy and thus were more likely than unvaccinated women to terminate the pregnancy, and unvaccinated women might have been more likely to terminate in the context of the outbreak. Also, STRIVE was launched during a terrible epidemic that caused enormous social upheaval. This timing might also have affected decision-making about pregnancy termination.

Vaccination with rVSVΔG-ZEBOV-GP has become an integral component of the public health response to recent Ebola outbreaks, including the ongoing outbreak in the Democratic Republic of the Congo (*3*,*26*), underscoring the urgency of obtaining a full understanding of the safety of the vaccine in pregnancy. The World Health Organization’s Strategic Advisory Group of Experts on Immunization, recognizing the high risk for maternal and fetal death from Ebola virus infection, has endorsed the need for careful evaluation of risks and benefits in a local context by national regulatory authorities and ethics committees in decision-making about rVSVΔG-ZEBOV-GP vaccination of pregnant women during an Ebola outbreak (*27*). The decision to offer rVSVΔG-ZEBOV-GP vaccine to pregnant women will need to balance the risk for an adverse pregnancy outcome with the risk for exposure to and subsequent infection with Ebola (*28*–*33*). When vaccination is offered to pregnant women, the provision of culturally appropriate information to assist women in making informed decisions about whether to accept vaccination will be critical. The STRIVE experience contributes information that should be useful for these decisions. It also highlights the urgent need for additional comprehensive and accurate pregnancy outcome information, whether through clinical trials, in which inclusion of pregnant women is increasingly being considered (*32*), or through observational strategies, such as data collection during outbreak response or pregnancy registries.
